# Morphological and Physical Analysis of Natural Phospholipids-Based Biomembranes

**DOI:** 10.1371/journal.pone.0107435

**Published:** 2014-09-19

**Authors:** Adrien Jacquot, Grégory Francius, Angelina Razafitianamaharavo, Fariba Dehghani, Ali Tamayol, Michel Linder, Elmira Arab-Tehrany

**Affiliations:** 1 Université de Lorraine, Laboratoire Ingénierie des Biomolécules, TSA 40602, Vandoeuvre-lès-Nancy, France; 2 Université de Lorraine, Laboratoire de Chimie Physique et Microbiologie pour l′Environnement, UMR 7564, Villers-lès-Nancy, France; 3 CNRS, Laboratoire de Chimie Physique et Microbiologie pour l′Environnement, UMR 7564, Villers-lès-Nancy, France; 4 Université de Lorraine, Laboratoire Interdisciplinaire des Environnements Continentaux, UMR 7360, Vandoeuvre-lès-Nancy, France; 5 CNRS, Interdisciplinaire des Environnements Continentaux, UMR 7360, Vandoeuvre-lès-Nancy, France; 6 School of Chemical and Biomolecular Engineering, the University of Sydney, Sydney, New South Wales, Australia; 7 Division of Biomedical Engineering, Department of Medicine, Brigham and Women's Hospital, Harvard Medical School, Cambridge, Massachusetts, United States of America; LAAS-CNRS, France

## Abstract

**Background:**

Liposomes are currently an important part of biological, pharmaceutical, medical and nutritional research, as they are considered to be among the most effective carriers for the introduction of various types of bioactive agents into target cells.

**Scope of Review:**

In this work, we study the lipid organization and mechanical properties of biomembranes made of marine and plant phospholipids. Membranes based on phospholipids extracted from rapeseed and salmon are studied in the form of liposome and as supported lipid bilayer. Dioleylphosphatidylcholine (DOPC) and dipalmitoylphosphatidylcholine (DPPC) are used as references to determine the lipid organization of marine and plant phospholipid based membranes. Atomic force microscopy (AFM) imaging and force spectroscopy measurements are performed to investigate the membranes' topography at the micrometer scale and to determine their mechanical properties.

**Major Conclusions:**

The mechanical properties of the membranes are correlated to the fatty acid composition, the morphology, the electrophoretic mobility and the membrane fluidity. Thus, soft and homogeneous mechanical properties are evidenced for salmon phospholipids membrane containing various polyunsaturated fatty acids. Besides, phase segregation in rapeseed membrane and more important mechanical properties were emphasized for this type of membranes by contrast to the marine phospholipids based membranes.

**General Significance:**

This paper provides new information on the nanomechanical and morphological properties of membrane in form of liposome by AFM. The originality of this work is to characterize the physico-chemical properties of the nanoliposome from the natural sources containing various fatty acids and polar head.

## Introduction

Liposomes are artificial lipid vesicles with a lipid membrane that can encapsulate, protect, and transfect active molecules such as proteins, nucleic acids, and drugs. This particular property allows the liposomes to be used as vectors or biological carriers in biomedicine, pharmaceutics (vectorization and drug delivery), genetics (transfection or gene transfer) and tissue engineering [1]. Liposome could be formed of much different class of lipids, but the main constituents of lipid membranes are phospholipids. Phospholipids are amphiphilic molecules containing water soluble, hydrophilic head section and a lipid-soluble, hydrophobic tail section. Their membrane is composed of one or more bilayers which create a lipophilic space. The innocuity of liposomes constituents and their ability for the encapsulation of both hydrophilic and lipophilic species make them ideal candidates for drug delivery [2], [3], [4].

Overcoming the poor solubility in water of lipophilic drugs is of particular interest to increase the drug availability and to reduce its negative side effects [5]. Liposome size, surface properties, and membrane fluidity can be modulated by changes in lipid composition or the addition of polymers, polysaccharides or specific specie to tune the drug delivery rate [6]. A key challenge is to predict encapsulation efficiency and release kinetics of drugs from liposomes based on their formulation. In the recent years, supported lipid bilayers have been studied by Atomic Force Microscopy (AFM) as model membranes [7]. Amongst the key advantages for the use of AFM in investigating soft-materials is the possibility to perform high-resolution imaging under conditions conducive to soft and biological materials such as in liquid and without applying vacuum [8]. For example, the influence of temperature, addition of surfactant, ions, polymers, adsorption/desorption kinetics, and biomolecules-membrane interactions have been evaluated by AFM imaging as well as force spectroscopy [9], [10], [11], [12], [13], [14]. AFM imaging provides information about domains formation in the bilayer and phase segregation depending on the physical state of lipids. It also permits to follow the evolution of membrane topography like the fluidizing, erosion, or strengthening of domains by the addition of new constituents [9], [15]. Bilayers topography at micrometer scale, and with a subnanometer resolution in height, can be completed with force spectroscopy experiments. The interaction force between tip and sample versus the tip-sample separation distance is measured which provides information about the mechanical properties of the bilayer. These mechanical properties depend on the lipid composition and lipid organization under the tip and permit the identification of segregated domains [16]. However, AFM has also proven to be effective as a nanoscopic tool of membranes, capable of performing measurements of mechanics, such as force and elasticity [17], [18] as well as a tool for nanomanipulation.

The aim of this work is to investigate the physico-chemical properties, organization, and nanomechanical stability of model and complex lipid bilayers from plant and marine sources. For these purposes, we use two different natural lecithin sources from rapeseed and salmon with complex mixes of phospholipids containing the saturated, monounsaturated and polyunsaturated fatty acids and two lipid model dipalmitoylphosphatidylcholine (DPPC) and dioleoylphosphatidylcholine (DOPC). Electrophoretic mobility, membrane fluidity and fatty acid composition are studied as physico-chemical properties. Supported phospholipid bilayers are prepared for the complex lecithin and the lipid model and studied by AFM, both in imaging and force spectroscopy. The influence of the phospholipid is observed on membrane properties.

## Materials and Methods

### Materials

Dipalmitoylphosphatidylcholine (DPPC) and dioleoylphosphatidylcholine (DOPC) were purchased from Avanti Polar Lipids (Alabastar, AL, USA). Phospholipids from rapeseed and salmon phospholipids were extracted by use of a low temperature enzymatic process in the absence of organic solvent [19]. CaCl_2_ and NaCl were purchased from VWR (Leuven, Belgium) and tris(hydroxymethyl)aminomethane (Tris) from Sigma Aldrich (St Louis, MO, USA). Buffers were prepared with ultrapure water and pH was adjusted with NaOH 1 M. They were systematically filtered on a 0.2µm filter (Minisart RC15, Sartorius, Germany). TMA-DPH was purchased from Molecular Probes (Invitrogen, Carlsbad, CA) and Muscovite Mica V1-Quality from Electron Microscopy Sciences (Hatfield, PA, USA).

### Fatty acid composition

Fatty acid methyl esters (FAMEs) were prepared as described by Ackman [20]. The separation of FAMEs was carried out on a Shimadzu 2010 gas chromatography (Perichrom, Saulx-lès-Chartreux, France) equipped with a flame-ionization detector. A fused silica capillary column was used (60 m, 0.2 mm i.d.×0.25 µm film thicknesses, SP™2380 Sopulco, Bellfonte-PA-USA). Injector and detector temperatures were set at 250°C. The column temperature was set initially at 120°C for 3 min, then rose to 180°C at a rate of 2°C/min and held at 220°C for 25 min. Standard mixtures (PUFA1 from marine source and PUFA2 from vegetable source; Supelco, Sigma–Aldrich, Bellefonte, PA, USA) were used to identify fatty acids. The results were presented as triplicate analyses.

### Lipid classes

Lipidic classes of salmon, rapeseed and soya lecithins were determined by Iatroscan (MK-5 TLC-FID, Iatron Laboratories Inc.,Tokyo, Japan). Each sample was spotted on ten Chromarod S-III silica coated quartz rods held in a frame. The rods were developed over 20 min in hexane/diethyl ether/formic acid (80∶20∶0.2, v:v:v), oven dried for 1 min at 100°C and finally scanned in the Iatroscan analyzer. The Iatroscan was operated under a hydrogen flow rate of 160 mL/min and air flow rate of 2 L/min. A second migration using a polar eluent of chloroform, methanol, and ammoniac (65∶35∶5, v:v:v) made it possible to quantify polar lipids. The FID results were expressed as the mean value of ten separate samples. The following standards were used to identify the sample components:

– Neutral lipids (1-monostearoyl-rac-glycerol, 1.2-dipalmitoyl-snglycerol, tripalmitin, cholesterol).
– Phospholipids (L-α-phosphatidylcholine, 3 *sn*-phosphatidylethanolamine, L-α-phosphatidyl-L-serine, L-α-phosphatidylinositol, lyso-phosphatidylcholine, sphingomyelin).

All standards were purchased from Sigma (Sigma–Aldrich Chemie GmbH, Germany). The recording and integration of the peaks were performed using the ChromStar internal software.

### Liposome preparation

Lipids were dissolved in chloroform and the organic solvent was removed by evaporation in a rotary evaporator. Lipids were resuspended in buffer (Tris 10 mM, NaCl 100 mM, CaCl_2_ 3 mM) to obtain a final concentration in lipid of 1 g/L. The solution was sonicated 2 minutes (1 s on, 1 s off) using a probe sonicator (SonicatorVibra cell 75115, 500 watt, Bioblock Scientific Co) at 40% of full power and filtered on a 0.2µm filter. The obtained solution was extruded using an Avanti Mini Extruder (Avanti Polar Lipids, Alabaster, USA) through a 0.1µm pore size polycarbonate filter (20 times, between 45 and 50°C, i.e. above the phase transition temperature of vesicles).

### Liposome size and electrophoretic mobility measurements

Liposome size distribution and electrophoretic mobility of liposomes were analyzed using Malvern ZetasizerNano ZS (Malvern instruments, UK) with DTS Nano software (6.12, Malvern Instruments, UK). Size measurements were performed by dynamic light scattering (DLS) with laser emitting at 633 nm. Liposome suspension was placed in a cylindrical cell (10 mm diameter) and the scattering intensity was measured at 25°C at a scattering angle of 173° relative to the emitted source. Intensity autocorrelation functions were analyzed by a CONTIN algorithm in order to determine the distribution of the translational z-averaged diffusion coefficient of the liposomes, *D_T_* (m^2^. s^−1^) thanks to equation (1):
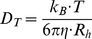
(1)


Where *k_B_* is the Boltzmann constant (in J.K^−1^), *T* is the temperature (K), η represents the fluid dynamic viscosity (Pa.s), and *R_h_* is the hydrodynamic radius of the liposome (m).

In suspension, particles are in a constant random Brownian motion, which causes fluctuations in the intensity of scattered light as a function of time. Refractive index was determined using a refractometer and at least 6 measurements were performed for each sample. The average values are presented along with standard error and polydispersity index (PDI). Electrophoretic mobility measurements were performed in standard capillary electrophoresis cells equipped with gold electrodes at 25°C. Liposome suspension was prepared in ultrapure water instead of buffer. Electrophoretic mobility mean values are presented with standard error.

### Preparation of supported lipid bilayers

Supported lipid bilayers (SLBs) were prepared by the vesicle fusion method according to Mingeot-Leclercq et al. (2008) [21]. Mica supports of 1×2 cm were glued on glass slides and mounted into atomic force microscopy (AFM) fluid cells. Mica sheets were freshly cleaved and the AFM fluid cells were placed into oven at 65°C for ten minutes. Then, 500µl of vesicle suspension in preparation buffer (Tris 10 mM, NaCl 100 mM, CaCl_2_ 3 mM) were deposed on mica sheets at 65°C. After 45 minutes, AFM fluid cells were filled with heated imaging buffer (Tris 10 mM, NaCl 100 mM) and cooled down to room temperature (1 hour). Then, mica supports were thoroughly rinsed with imaging buffer avoiding any contact of the SLB with air. Finally, fluid cells were placed in the AFM device for the experimentations.

### Atomic force microscopy imaging

Images of the different supported lipid bilayers (SLBs) were acquired on a Bruker AFM Dimension FastScan (Bruker, Billerica, MA, USA) with NPG tips (Bruker, Billerica, MA, USA) with spring constant of about 0.32 N/m (manufacturer data). Images were obtained at room temperature in Peak Force QNM mode, shortly after SLB formation. Images of 5, 10 and 20µm size were acquired at least for two different samples and two different areas per sample. Images were analyzed using Nanoscope Analysis (v140r2). Depth analyses as well as profile analyses were made on each image to determine bilayers height and one section is presented per image.

### Force spectroscopy measurements

Atomic force spectroscopy experiments were performed in force-volume mode on an Asylum MFP-3D Bio (Santa Barbara, CA, USA) with NPG tips (Bruker-AXS, Palaiseau, France). Cantilever spring constant was calibrated thanks to the thermal noise method. [22] Approach-retract cycles were performed on 32×32 grids of 5×5 µm^2^ for bilayers made of rapeseed phospholipids, salmon phospholipids, DOPC, and DPPC. Typical force curves are shown presenting a rupture corresponding to the breakthrough of the bilayer. Each curve was treated using Matlab (R2010b, Mathworks Inc., Natick, MA, USA) through an algorithm developed in the lab. Briefly, deflection sensitivity was calibrated on a hard surface (glass) and breakthrough events were detected on force curves to determine the rupture force. The contact point was first approximated and then determined as well as the elastic modulus of the sample by fitting the approach curves with a Sneddon model [23]:
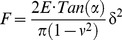
(2)


Where *F* is the applied force (N), *E* the elastic modulus (Pa), α the tip half-angle, ν the Poisson's ratio (assumed to be 0.5) and δ the indentation or a the deformation depth on the samples surface (m). The obtained values of rupture force and elastic modulus were presented in histograms and the distribution was fitted with a Gaussian under Origin 6.1 (OriginLab, MA, USA). Results are presented as distribution center ± half-width.

### Membrane fluidity

Membrane fluidity of liposome for the various compositions was determined by fluorescence anisotropy measurements using a Tecan INFINITE 200^R^ PRO (Austria). Trimethylammonium-diphenylhexatriene was used as fluorescent probe [24] with TMA improving the localization of the probe in surface interior. A solution of TMA-DPH 1 mM in ethanol was prepared and added to liposome suspension to reach a final concentration of 4 µM. The suspension was gently stirred for an hour. For fluorescence anisotropy measurements, 200 µL of liposome suspension are distributed in a non-binding black microplate (Greiner Bio-One, Germany) and are excited under a polarized laser at the excitation wavelength of 360 nm. The fluorescence intensity is measured in the parallel and perpendicular planes of polarization at 430 nm. Measurements are carried on at 25°C under stirring. Fluorescence anisotropy is calculated using the following equation, 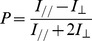
(3)where I_//_is the intensity in the plane parallel to excitation plane and I_⊥_ the fluorescence intensity in the plane perpendicular to excitation plane. The fluorescence anisotropy has a direct relationship with I_//_/I_⊥_, *i.e.*, when the reorientation time of the fluorescent probe is bigger. Membrane fluidity is defined as 1/P and was calculated with 8 measurements per sample.

### Statistical analysis

All data are presented as mean ± standard error. Statistical significance was determined by one-way Anova tests with p value lower than 0.05 using SigmaPlot software 11.0.

## Results and Discussion

### Fatty acid composition and lipid classes

Fatty acid compositions of phospholipids from rapeseed and salmon are presented in **Table 1**. It can be seen that polyunsaturated fatty acids of salmon count for 50% of the composition, especially DHA (C22:6n3) and EPA (C20:5n3) compared to rapeseed phospholipids. We also observed the low ratio of saturated fatty acids in phospholipids from rapeseed (9%) compared to that of salmon (27%) and the predominance of monounsaturated fatty acid, especially C18:1n9 (57%) for rapeseed phospholipids. The most abundant constituent of rapeseed lecithin was a monounsaturated fatty acid and C18:3 n-3 had the highest concentration among polyunsaturated fatty acids (6.60%). The most present monounsaturated fatty acid in salmon lecithin was C18:1 n-9 (56.51%) while, C22:6 n-3 and C20:5 n-3 were counted for 23.81% and 9.56% of polyunsaturated fatty acids, respectively. The ratio of n-3/n-6 was higher in salmon lecithin compared to rapeseed lecithin with 3.80, and 0.25, respectively.

**Table 1:** Main fatty acid composition of plant and marinephospholipids by gas chromatography (area%).

Fatty acids	Salmon phospholipids	Rapeseed phospholipids
C14:0	1.59±0.01	-
C15:0	0.22±0.01	-
C16:0	16.21±0.01	7.41±0.01
C17:0	0.45±0.09	-
C18:0	4.67±0.06	1.31±0.00
C20:0	-	0.36±0.01
C21:0	1.90±0.01	-
C22:0	0.78±0.01	0.21±0.02
C23:0	1.22±0.02	-
**SFA**	**27.03**	**9.28**
C15:1	0.48±0.31	-
C16:1	1.46±0.08	0.33±0.01
C17:1	1.21±0.10	-
C18:1n9	19.58±0.02	56.51±0.04
C20:1n11	0.30±0.02	0.72±0.04
C22:1n9	-	0.25±0.03
**MUFA**	**23.03**	**57.80**
C18:2n6	5.75±0.03	26.32±0.04
C18:3n3	2.68±0.01	6.60±0.01
C20:2n6	0.29±0.02	-
C20:3n6	0.28±0.04	-
C20:3n3	0.34±0.10	-
C20:4n6	2.43±0.08	-
C20:5n3 (EPA)	9.56±0.06	-
C22:4n6	1.62±0.02	-
C22:5n3	3.27±0.06	-
C22:6n3 (DHA)	23.81±0.29	-
**PUFA**	**49.94**	**32.91**
n-3/n-6	3.80	0.25
DHA/EPA	2.51	-

The lipid classes of lecithins were separated by thin-layer chromatography (Iatroscan). However, the percentage of polar fraction showed that rapeseed phospholipid was richer in polar lipids with 75.5±0.9% in compared to salmon phospholipid with 62.3±0.8%.

### Electrophoretic mobility and size measurements

Electrophoretic mobility and size measurements are important to evaluate liposomes size and stability in aqueous medium. A high value of electrophoretic mobility leads to a repulsion between liposomes, which can prevent their aggregation and is a measure of liposomal stability. Values of electrophoretic mobility obtained for nanoliposomes made of rapeseed, salmon phospholipids, DOPC, and DPPC are presented in **Table 2**. We found a significant higher value of electrophoretic mobility for DOPC compared to DPPC (*P*<0.05) and much higher mobility for liposomes made of rapeseed and salmon phospholipids (>3 µm.cm. V^−1^.s). The results suggest that nanoliposomes were stable and the negative value of electrophoretic mobility was due to the presence of negatively charged phospholipids. However, we found the higher electrophoretic mobility for liposomes made of rapeseed phospholipids, which were the largest liposomes (125 nm) compared to that of DOPC (102 nm), DPPC (103 nm) or salmon phospholipids (57 nm).

**Table 2:** Electrophoretic mobility of liposomes.

Nanoliposome composition	Electrophoretic mobility (µmcm/Vs)
DOPC	−0.89^a^±0.08
DPPC	−0.47^b^±0.04
Rapeseed phospholipids	−3.54^c^±0.16
Salmon phospholipids	−3.44^c^±0.08

Data were expressed as mean ± SD (n = 6).

(Significant difference between a, b and c; P<0.05).

Size of nanoliposome is crucial parameter for drug delivery across biological barrier or drug encapsulation and depends on lipid composition as well as liposome preparation method. Size measurements were carried for the various mixtures between DOPC, DPPC and rapeseed or salmon phospholipids (**Table 3**). The values were around 100 nm for liposomes made of DOPC, DPPC or a mixture between DOPC and DPPC (1∶1). The smallest liposome was formed from salmon phospholipids with a z-average around of 57 nm. The addition of DOPC or DPPC to salmon phospholipids (1∶1) increased the size of liposomes to 64 and 65 nm, respectively. Addition of DOPC to rapeseed phospholipids yielded a slight decrease from 125 to122 nm, whereas it rises to 132 nm with the addition of DPPC. We can observe for ternary mixture based on salmon phospholipids an increase of size with increasing the proportion of salmon phospholipids from 71 to 104 nm, which does not tend to the value of 57 nm found for liposomes made of salmon phospholipids only. Results for ternary mixtures based on rapeseed phospholipids shows a significant increase of liposome size from 119 to 141 nm with increasing the rapeseed phospholipids ratio from 50 to 80%.

**Table 3:** Size and membrane fluidity of liposome made of various phospholipids mixture by dynamic light scattering and fluorescence anisotropy.

Nanoliposome composition	Size (nm)	PDI	Membrane fluidity
DOPC	102.3^ab^±1.0	0.13±0.01	9.94±0.51^a^
DPPC	102.9^a^±0.8	0.04±0.01	5.04±0.05^b^
DOPC + DPPC (1∶1)	100.1^b^±1.2	0.15±0.01	8.19±0.45^c,d^
Rapeseed phospholipids	125.2^c^±1.0	0.14±0.01	8.45±0.26^c,h^
Salmon phospholipids	57.1^d^±0.4	0.10±0.01	7.70±0.21^e^
Rapeseed ph. + DOPC (1∶1)	122.2^e^±2.1	0.11±0.02	9.48±0.42^f^
Rapeseed ph. + DPPC (1∶1)	131.8^f^±2.1	0.16±0.01	8.40±0.13^c,d,h^
Salmon ph. + DOPC (1∶1)	63.9^g^±0.5	0.11±0.01	8.78±0.21^g^
Salmon ph. + DPPC (1∶1)	65.1^g^±0.3	0.08±0.01	6.11±0.11^i^
Rapeseed ph. + DOPC + DPPC (1∶1∶1)	118.8^h^±1.5	0.14±0.01	8.57±0.18^g,h^
Rapeseed ph. + DOPC + DPPC (2∶1∶1)	118.5^h^±1.6	0.22±0.01	8.61±0.17^g,h^
Rapeseed ph. + DOPC + DPPC (4∶1∶1)	135.2^i^±0.6	0.13±0.01	9.24±0.30^f,k^
Rapeseed ph. + DOPC + DPPC (8∶1∶1)	140.9^j^±1.4	0.15±0.01	9.12±0.49^k^
Salmon ph. + DOPC + DPPC (1∶1∶1)	71.4^k^±0.9	0.10±0.01	7.76±0.23^e^
Salmon ph. + DOPC + DPPC (2∶1∶1)	71.1^k^±0.7	0.09±0.01	7.68±0.25^e^
Salmon ph. + DOPC + DPPC (4∶1∶1)	97.7^l^±3.5	0.29±0.03	8.12±0.18^d^
Salmon ph. + DOPC + DPPC (8∶1∶1)	103.8^a^±1.8	0.28±0.01	8.49±0.43^c,g^

Data were expressed as mean ± SD (n = 6).

(Values with a common letter are not significantly different (P<0.05)).

### AFM imaging

**Figure 1:**
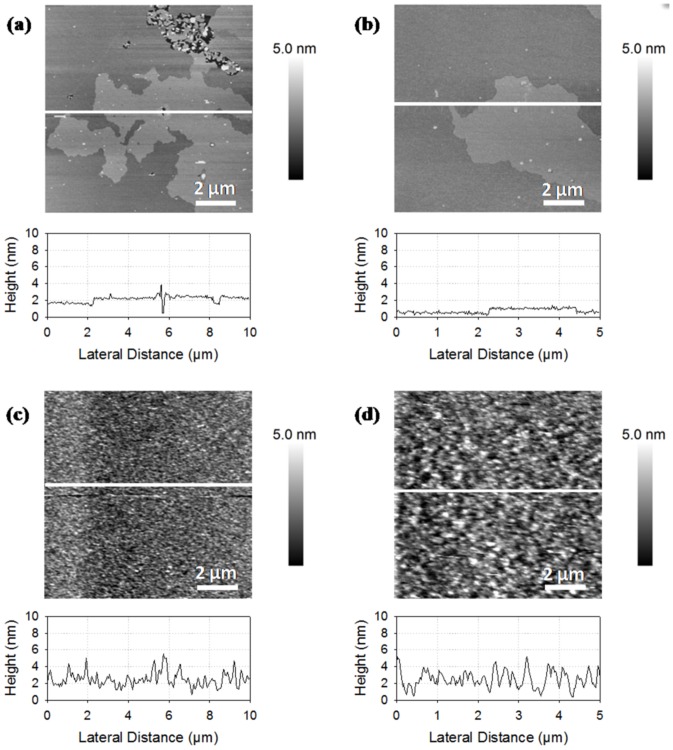
AFM images of supported lipid bilayers made of phospholipids from rapeseed (a, b) and from salmon (c, d) presented with a profile of a section taken on the image (white lines).

**Figure 2:**
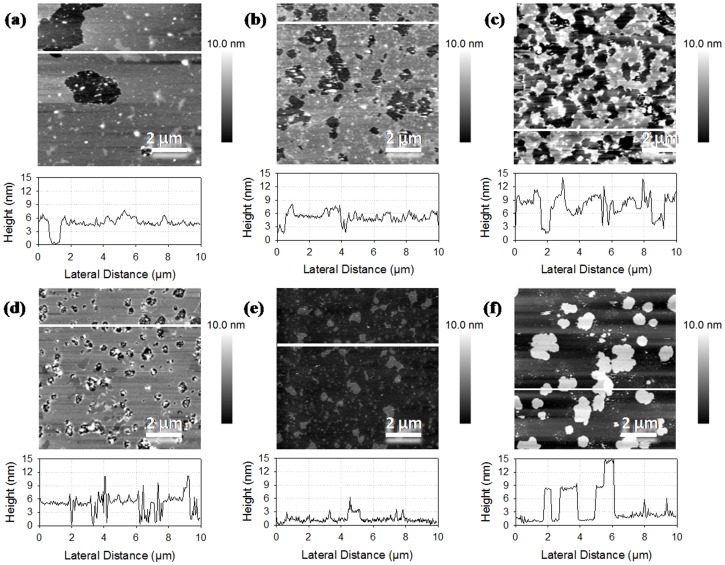
AFM images of supported lipid bilayers made of phospholipids from rapeseed mixed with DOPC and DPPC. Ratios of rapeseed phospholipids, DOPC, and DPPC are respectively 1∶1∶0 (a), 1∶0∶1 (b), 1∶1∶1 (c), 2∶1∶1 (d), 4∶1∶1 (e), and 8∶1∶1 (f).

AFM images provide the topographical organization of supported lipid bilayers at the micrometer scale with a subnanometer resolution in height. The morphology of rapeseed and salmon phospholipids membranes are shown in **Figure 1**. The membrane made of phospholipids from rapeseed exhibited two separated domains with height differences of 0.7±0.2 nm (**Figure 1a and 1b**). This value appears lower than the height difference of 1 nm described in the literature for phase segregation of DPPC and DOPC [25]. Contrary to membranes made of phospholipids from rapeseed, those of phospholipids from salmon did not show phase segregation with the occurrence of gel-phase domains of several squared micrometers (**Figure 1c and 1d**). Only a disorganized layer characterized by a heterogeneous thickness was observed (height profiles showing differences ranging from 0 to 5 nm). AFM images confirmed the presence of lipid deposit onto the mica surface, but did not prove that disorganized structure was a supported lipid bilayer. This assumption was tested by force spectroscopy experiments. The phospholipids composition for salmon was complex with an important part of polyunsaturated fatty acids that may explain the good mixing of phospholipids yielding in relatively high height differences on topographical images. In order to have references and to get information about plant and marine membranes organization, we mixed plant and marine phospholipids with DOPC and DPPC, two pure phospholipids previously studied by AFM [7], [26], [27]. These pure phospholipids are in two different physical states at room temperature, in liquid disordered state and in gel like state for DOPC and DPPC, respectively. Comparing the mixture of DOPC/rapeseed phospholipids (1∶1), from **Figure 2a** to **Figure 1a**, we can observe larger holes across a main bilayer. Small and scrappy domains are dispersed over the main bilayer with height differences of about 2 nm. This difference of height was lower than the total thickness of DOPC bilayer but was twice of the observations on DPPC/DOPC membrane and described in the literature with 1.1 nm [28]. DOPC is composed of two chains of C18:1n9, the predominant fatty acid in rapeseed phospholipids, and we expected the formation of a common phase. The increase in height differences between domains from 0.7 to 2.0 nm suggests a fluidizing of the membrane from rapeseed phospholipids by the addition of DOPC resulting in thickness reduction of the bilayer.

**Figure 2b** shows an AFM image of the membrane based on the mixture of DPPC and rapeseed phospholipids (1∶1). As with DOPC, the mica support was not fully covered by the bilayer, which formed two domains with a height difference of 1.1 nm. Domains were more dispersed in the mixture with DPPC compared to the mixture with DOPC. These observations may be due to the presence of ordered domains of DPPC in a fluid matrix of rapeseed phospholipids. **Figures 2c to 2f** shows the AFM images of liposomes formed from mixtures of rapeseed phospholipids, DOPC, and DPPC with various ratios (ratio from 1∶1∶1 to 8∶1∶1). The bilayer covered the majority of the mica support with the increase of the rapeseed phospholipids ratio. The surface coverage rate increased with the ratio of rapeseed phospholipids in the mixture (Figures **2c** to **2f**). The surface was fully covered on images **2e** and **2f** and domains were formed over the main/below membrane. We hypothesize that a fluid composition of rapeseed phospholipids and probably DOPC should cover the mica surface and expelling DPPC bilayers and thus avoid the formation of gel-phase domains.

**Figure 3:**
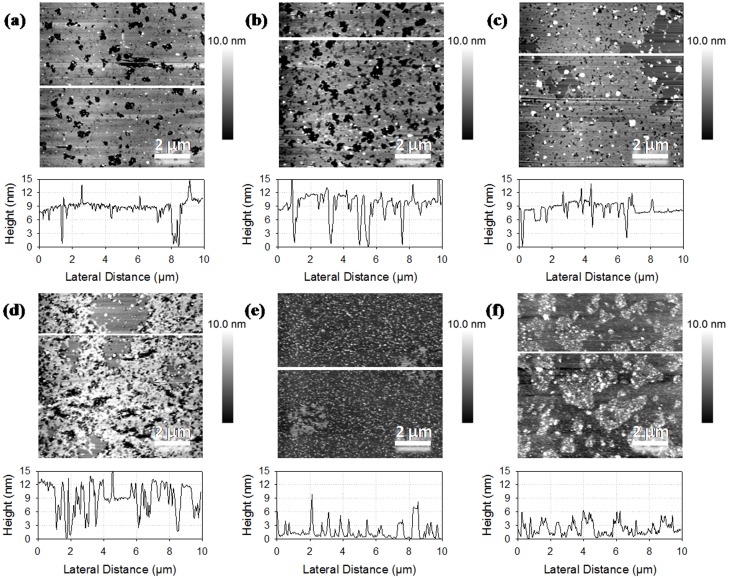
AFM images of supported lipid bilayers made of phospholipids from salmon mixed with DOPC and DPPC. Ratios of salmon phospholipids, DOPC and DPPC are respectively 1∶1∶0 (a), 1∶0∶1 (b), 1∶1∶1 (c), 2∶1∶1 (d), 4∶1∶1 (e), and 8∶1∶1 (f).

Images in **Figure 3a** to **3d** show the domains segregations with bilayer values around 9 nm corresponding to bilayer stacking. In **Figure 3c**, we observe important flat domains and the rest of the image is similar to that of DOPC mixed with rapeseed phospholipids, which indicates that a fluid phase of rapeseed phospholipids and DOPC covered DPPC ordered domains. It also suggests the heterogeneity when mixing salmon phospholipids with DOPC or DPPC. Height differences of about 2.7 nm and holes are observables on **Figure 3a**. By comparing Figures **3a and 3b**, it can be concluded that the mix was less heterogeneous with DOPC, which might have thinned the salmon membrane whereas DPPC forms segregated domains. However, DPPC domains organized the membrane for salmon phospholipids too, comparing **Figures 1c and 1d**. Images in Figures **3e and 3f**, for mixes with more than 50% of salmon phospholipids, were similar to membrane made of salmon phospholipids only with a rough surface. We observed some patches of 3 or 6 nm that might have been attributed to the exclusion of more ordered domains from salmon phospholipids membrane. Comparing the addition of pure phospholipids in rapeseed and salmon phospholipids mixtures, we observed similar tendencies of segregation of gel like phase domains and a good association with DOPC at significant ratios of pure phospholipids, whereas the surface coverage increases and gel like phase domains were excluded of the bilayer at lower ratios of pure phospholipids.

### Membrane fluidity

Membrane fluidity was determined for each mixture between DOPC, DPPC, and plant or marine phospholipids. Membrane fluidity is related to the lateral fluctuation of lipids in the membrane and plays a role in transduction of molecules across the membrane. The presence of unsaturated fatty acid tends to increase membrane fluidity in comparison with saturated fatty acids [29]. The values of membrane fluidity were the highest and the lowest for liposome made of DOPC and DPPC, respectively. That can be explained by two insaturations bonds in DOPC fatty chains, whereas those of DPPC are saturated, resulting in lower chain mobility. We determined an intermediate membrane fluidity for liposomes made of salmon and rapeseed phospholipids. According to the measured data, membrane fluidity decreased with the proportion of saturated fatty chains in the lipid composition of liposome. Membrane fluidities of liposome made of DOPC, DPPC, rapeseed phospholipids and salmon phospholipids were significantly different (*P*<0.05).

For binary mixture, the addition of DOPC to rapeseed phospholipids or salmon phospholipids yields to an increase of liposome membrane fluidity whereas the addition of DPPC decreases it. Membrane fluidity values for ternary mixtures with rapeseed phospholipids, DOPC, and DPPC with the ratios 1∶1∶1 and 2∶1∶1 were not significantly different (*P*<0.05) from the membrane fluidity value of rapeseed phospholipids alone. However, we observe a significant increase in membrane fluidity for ternary mixtures with the ratios 4∶1∶1 and 8∶1∶1. The same observation was made for ternary mixtures based on phospholipids from salmon.

### Atomic force spectroscopy

**Figure 4:**
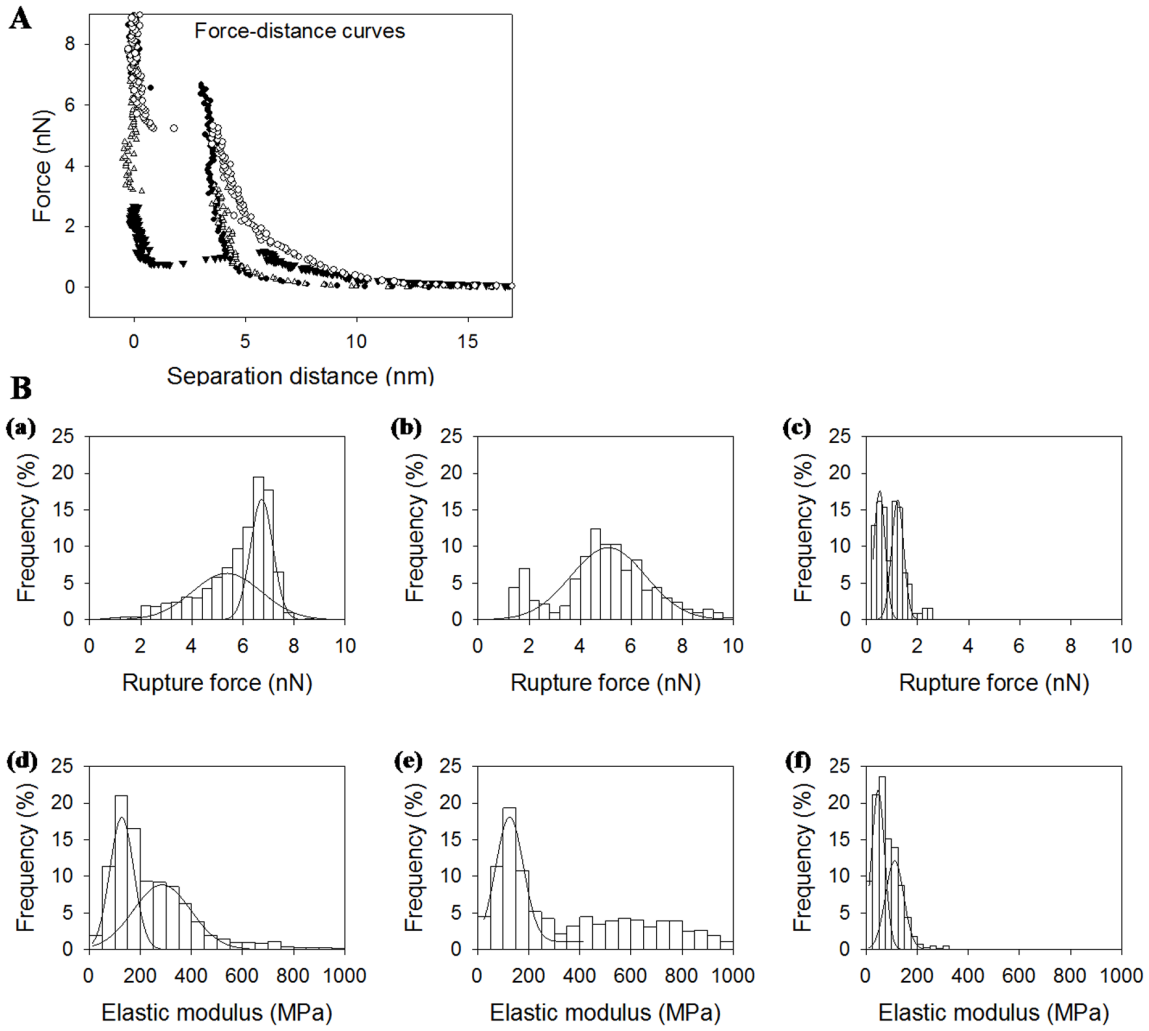
(A). Typical force-distance curves for bilayers made of DPPC (black circles), DOPC (empty triangles), rapeseed phospholipids (empty circles) and salmon phospholipids (black triangles). (B). Distribution of rupture forces (up) and elastic moduli (down) for bilayers made of DOPC/DPPC (a,d), salmon phospholipids (b,e), rapeseed phospholipids (c,f)

Force-distance curves were acquired for supported lipid bilayers made of DOPC and DPPC (1∶1), rapeseed phospholipids and salmon phospholipids. Typical force-distance curves are reported in **Figure 4**. For separation distances over 20 nm, no interaction took place and the tip-sample interaction force was null. By approaching the tip closer to the sample, the force increased through long range interactions and then through the indentation of the sample. The force needed to indent the bilayer increased with tip penetration until it reached a maximum in force. At this point, the bilayer was broken under tip indentation, and the tip came closer to the mica support. The maximal force before bilayer's rupture was characteristic from the lipid composition and lipid organization of the bilayer. It is like a fingerprint of the bilayer and according to Attwood et al. (2013) and Garcia-Manyes and Sanz (2010) [27], [30], which is related to the lateral fluctuation of lipids and depends on the physical state of the lipids under the tip. Distributions of the rupture forces found are presented **Figure 4B** **(a, b,c)** and were fitted with Gaussian curves. Distribution center and half-width according to the fit are presented in **Table 4**. The force needed to break the bilayer made of salmon phospholipids was the lowest in comparison to rapeseed phospholipids, DOPC, and DPPC. We attribute the distribution of highest rupture forces in the mixture of DOPC and DPPC to DPPC domains because of lipids physical state according to Picas et al. (2012) [31]. Bilayer's rupture force for DOPC was similar to that for rapeseed phospholipids around 5 nN. The high proportion of polyunsaturated fatty acids in salmon phospholipids might have been the reason for the easier tip penetration in the bilayer.

**Table 4:** Rupture forces and elastic moduli of different membranes by atomic force spectroscopy.

Membrane composition	Rupture force (nN)			
DOPC + DPPC	5.3±1.4	54%	6.7±0.5	46%
Rapeseed phospholipids	5.1±1.5	100%		
Salmon phospholipids	0.5±0.2	50%	1.2±0.3	50%
	**Elastic modulus (MPa)**			
DOPC + DPPC	128±47	46%	285±113	54%
Rapeseed phospholipids	114±28	100%		
Salmon phospholipids	47±26	56%	112±36	44%

Data were expressed as mean ± half-sigma (n = 1024).

The force-distance curve was also fitted before the breakthrough event with an elastic model for tip indentation in order to determine the elastic modulus of the sample depending on the force needed to compress the bilayer. As the contact point was not clearly defined in AFM force-distance curves, an approximate contact point and elastic modulus were first determined. Then, the values were modified around the first estimated value in order to find the best fit. The distributions of the determined elastic modulus values are presented in **Figure 4B** **(d, e, f)** and the results of Gaussian fit in **Table 4**. Furthermore, we attributed the highest value of elastic modulus to DPPC domains for the DOPC plus DPPC mix. We observed a bimodal distribution of elastic modulus for salmon phospholipids membrane with centers around 47 and 122 MPa. We found similar values of elastic modulus for DOPC and rapeseed phospholipids membranes considering distributions width (128 and 114 MPa respectively). Considering AFM images, we could expect a monomodal distribution for salmon phospholipids and a bimodal one for rapeseed phospholipids. For rapeseed membranes, the differences in mechanical properties were too slight to be distinguished in our experiments. The bimodal distribution for salmon membrane may be found its origin in the low number of lipids under the tip during approach retract cycles and the possibility to probe patches of only hundreds square nanometers.

## General Discussion and Conclusion

The understanding of the contribution from different constituents in phospholipids membranes remains challenging. Biological membranes are complex assemblies of carbohydrates, proteins and lipids essentials in biological activity and are exposed to various external conditions. These membranes present heterogeneities at the micro- or nanoscale where the diffraction limit hinders optical studies. Atomic force microscopy and its derivatives are interesting tools to study membranes at this scale and in a liquid environment.

In this study, complex mixtures of phospholipids from natural resources were analyzed to assess the role of lipid composition on membrane properties. The fatty acid composition of phospholipids from rapeseed and salmon were analyzed and liposomes in buffer were obtained by sonication and extrusion techniques. We studied the membrane properties by employing the fluorescence anisotropy measurements for membrane fluidity determination. Supported lipid bilayers obtained from liposomes were analyzed by atomic force microscopy for topographical imaging as well as force spectroscopy to probe membranes mechanical properties. DOPC and DPPC were used as references for the different analyses and were mixed to plant and marine phospholipids obtained by green process to observe membrane evolution following lipid composition changes.

The inspection of particle size measurements results showed that liposomes have average sizes in the range from 50 to 150 nm as a function of phospholipid sources. Liposomes from rapeseed phospholipids showed an average size more than two times bigger than liposomes made from salmon phospholipids, but electrophoretic measurements do not show a significant difference. Compared to liposomes made of DOPC or DPPC, electrophoretic mobility for liposomes made of plant or marine phospholipids were stronger and all were negatives due to negatively charged phospholipids. Our observations by atomic force spectroscopy imaging showed a high heterogeneity of rapeseed phospholipids membranes with two segregated domains, whereas salmon phospholipids are fully dispersed in the membrane. DPPC domains were formed in mixes without getting loose by rapeseed or salmon phospholipids, whereas DOPC may thin the membranes of plant or marine phospholipids in equivalent proportion. We observed a change in membrane topographical images, membrane fluidity and size measurements for liposomes made of ternary mixtures, *i.e.* DOPC, DPPC, and rapeseed or salmon phospholipids. Size and membrane fluidity of liposomes increases for mixtures with 4∶1∶1 and 8∶1∶1 ratios compared to the other ones and domains were excluded over the membrane in topographical images. A small addition of DOPC and DPPC to rapeseed or salmon phospholipids did not form a good mixture. The results obtained by force spectroscopy showed that membrane made of rapeseed phospholipids had similar mechanical properties to that of DOPC bilayer and weaker than DPPC bilayer, whereas salmon phospholipids membrane had the weaker mechanical properties. This may be due to the high proportion of polyunsaturated fatty acids in salmon phospholipids, making tip penetration favorable, but this observation was in contradiction with the membrane fluidity of liposomes determined by fluorescence anisotropy, which was lower for salmon phospholipids than rapeseed phospholipids or DOPC. Indeed, fluorescence anisotropy measurements indicated a decrease in liposomes membrane fluidity with increasing of saturated fatty acids. The questions remains open and we can maybe formulate the hypothesis of a preferential anchoring of the fluorescent probe TMA-DPH in the most organized part of the membrane.

For the first time, we showed the organization of complex phospholipid membrane made from natural sources with a high variety of lipid compared to previous studies on model membranes. Lipid composition modified liposomes properties, which have been investigated by atomic force microscopy and spectroscopy. It demonstrates the importance of fatty acid composition for membrane properties and gives insights for liposomes formulations. Besides lipid composition, the addition of polymers, polysaccharides, proteins or else as well as external middle conditions, remain determinants factors. Following studies will focus on the objective of carrying hydrophobic drugs in liposome membrane to overcome their poor solubility and stability in water and in order to control their delivery. The interaction of drug with the membrane yields in structure evolution and membrane properties changes that could be studied by the techniques presented in this work that could be complemented by other techniques focusing on the bilayer-drug interaction at a single molecule level. It will be kept in mind that drug encapsulation efficiency and drug release will be influenced by lipid composition and has to be measured.
